# Impact of Alginate Composition: From Bead Mechanical Properties to Encapsulated HepG2/C3A Cell Activities for *In Vivo* Implantation

**DOI:** 10.1371/journal.pone.0062032

**Published:** 2013-04-25

**Authors:** Stephanie H. Capone, Murielle Dufresne, Mathias Rechel, Marie-José Fleury, Anne-Virginie Salsac, Patrick Paullier, Martine Daujat-Chavanieu, Cecile Legallais

**Affiliations:** 1 UMR CNRS 7338, Laboratory of Biomechanics and Bioengineering, University of Technology, Compiegne, France; 2 INSERM U1040, CHU St Eloi, Institute of Research in Biotherapy, Montpellier, France; Faculty of Medicine University of Leipzig, Germany

## Abstract

Recently, interest has focused on hepatocytes’ implantation to provide end stage liver failure patients with a temporary support until spontaneous recovery or a suitable donor becomes available. To avoid cell damage and use of an immunosuppressive treatment, hepatic cells could be implanted after encapsulation in a porous biomaterial of bead or capsule shape. The aim of this study was to compare the production and the physical properties of the beads, together with some hepatic cell functions, resulting from the use of different material combinations for cell microencapsulation: alginate alone or combined with type I collagen with or without poly-L-lysine and alginate coatings. Collagen and poly-L-lysine increased the bead mechanical resistance but lowered the mass transfer kinetics of vitamin B12. Proliferation of encapsulated HepG2/C3A cells was shown to be improved in alginate-collagen beads. Finally, when the beads were subcutaneously implanted in mice, the inflammatory response was reduced in the case of alginate mixed with collagen. This *in vitro* and *in vivo* study clearly outlines, based on a systematic comparison, the necessity of compromising between material physical properties (mechanical stability and porosity) and cell behavior (viability, proliferation, functionalities) to define optima hepatic cell microencapsulation conditions before implantation.

## Introduction

In severe cases of hepatic failure, orthotopic or auxiliary liver transplantations are required for patient’s survival. Today, these therapies are considered as the only effective ones to restore liver functions and to improve the patient’s survival. Additional strategies are required to significantly reduce the patient’s death rate [1]. Extensive research work and limited clinical trials have shown that hepatocyte transplantation into the liver or in ectopic sites may be useful to bridge some patients to orthotopic liver transplantation [2], [3]. In the cases of intraportal or intrasplenic infusion, major complications can occur, such as portal vein thrombosis, portal hypertension and pulmonary embolism [4]. Moreover, feasibility studies outlined that a significant amount of implanted hepatocytes is lost during the first hours after the implantation due to the stringent conditions imposed to the cells by the blood flow [5]. Lastly, use of allogenic hepatocyte transplantation requires, as whole liver transplantation, chronic immunosuppressant treatment to avoid rejection.

Cell encapsulation in a porous biomaterial represents an alternative to overcome some of these drawbacks. The encapsulation process retains the cells in a semi permeable porous structure or membrane that both protects them from damage by the host immune system and maintains survival and metabolic functions, allowing the bidirectional transfer of oxygen, nutrients and cellular products. Numerous studies have been conducted to treat liver failure in animal model with microencapsulated hepatic cells [6]–[7]. The carbohydrate polymer Na-alginate (Na-alg) is a biocompatible material extracted from the cell walls of brown algae. Na-alg is a block-wise copolymer of α-L-guluronic acid (G-block) and 1,4-linked β-D-mannuronic acid (M-block) [8], the first one offering an affinity to certain bications, as Ca^2+^, Ba^2+^ or Sr^2+^ [9]. During the gelation process, G-G and M-G homo- or heteroblocks are electrostatically cross-linked with divalent ions and form a porous matrix, e.g. Ca-alginate (Ca-alg). Mechanical properties of Ca-alg (rigidity, stiffness, swelling) can be tuned by physical factors such as cross-linking density, cross-linker type, molecular weight distribution and viscosity of extracted Na-alg, as well as the overall ratio M-block/G-block or by chemical modification of the polymer Na-alg. Ca-alg hydrogel porosity can be adjusted to be permeable to small and essential molecules (gas, glucose or albumin) but not to larger ones, such as immunoglobulin G [10]. If alginate salts are well recognized for their biocompatibility and provide a hydrated matrix for cell encapsulation [11], anchorage-dependent cells cannot attach and spread on alginate surface, limiting biological interaction between them and hydrogel [12].

Among approaches developed to control liver-specific function expression, a microenvironment enriched in collagen, such as double gel configuration [13] or collagen coated dishes [14], has shown its efficiency. Collagen is a major protein component of extracellular matrix which presents the capability of specific cell interactions. Adhesion to collagen regulates hepatocyte behavior through transmembrane adhesion receptors such as integrins. Dependent upon the collagen form, fibrillar or monomeric, hepatocyte adhesion to type I collagen can elicit different cellular responses. When polymerized into fibrils, a configuration similar to the *in vivo* one, type I collagen gel maintains a rounded morphology, promotes hepatocyte long term survival and increases differentiated functions [15]. In spite of its biological properties, only few reports described the hepatocyte encapsulation in a mixture alginate - collagen. Collagen, used for hepatocyte encapsulation in beads or capsules, is mostly elaborated as gel or nanofibres surrounded by a polymeric membrane [16]–[17]. Collagen fibres can then be modified to provide physical and chemical properties allowing optimal hepatocyte functions. Aoki demonstrated the advantage of this 3D environment composition on rat hepatocyte survival and functions *in vitro*, just as an improvement of liver functions after intrasplenic transplantation of microencapsulated hepatocytes in fulminant hepatic failure in a rat model [6].

Another limit of pure alginate concerns its relative instability due to the nature of ionic liaisons between alginate residues. In physiological or culture conditions, divalent ions are exchanged for monovalent ones, decreasing mechanical resistance of alginate matrix. To enhance the resistance of alginate, beads can be coated with different molecules. For instance, poly-L-lysine (PLL) is a natural biopolymer with a high interaction with the alginate G-blocks. A PLL layer on alginate beads was shown to increase the bead resistance [18]. As PLL is an inflammatory molecule responsible for fibrotic overgrowth [18]–[20], an external layer of alginate can be added to mask this effect [21]. This double surrounding layer has demonstrated its efficiency to prevent cell leakage from hydrogel beads, which is, particularly in a transplantation application, of great importance [22].

In the present study, we encapsulated hepatic cells in an alginate matrix enriched or not with type I collagen and covered or not with a PLL layer. Literature data did not allow a full comparison between collagen and PLL added to alginate beads, especially regarding all the requirements listed for efficient encapsulated hepatic cell transplantation [23]. Then we systematically analysed the influence of the various Ca-alg based matrix compositions on the cell encapsulation process, as well as on the mechanical resistance and diffusion properties of the resulting beads. The biological behavior of human hepatocarcinoma cells (HepG2/C3A) encapsulated within the beads was compared over 10 days of culture. This cell line was widely employed in the literature as a relevant cellular model for hepatocyte despite some metabolic limitations [24], [25] and allowed us to focus on the biomaterials’ composition effect, limiting the large variability inherent to the use of human primary hepatocytes collected from different donors. The use of HepG2/C3A cells is also authorized for phase III clinical trials of the ELAD liver support device [1]. In other respects, the configuration of HepG2/C3A cells encapsulated in beads has emphasized the advantages on their behavior of maintaining these cells in an alginate 3D environment, regulating significantly higher expression of differentiated functions [26], [27]. Alginate-encapsulated HepG2/C3A cells have improved some systemic parameters of liver failure in a rabbit model [28] and sustained metabolic, synthetic and detoxificatory activities in human liver failure plasma [29].

Finally, we addressed the question of biocompatibility towards the host, following subcutaneous implantation of empty beads in immunocompetent mice. These data are discussed in closed link with the guidelines of encapsulated hepatic cell transplantation.

## Materials and Methods

### Chemicals

Sodium chloride, HEPES, calcium chloride, citrate sodium, sodium alginate (medium viscosity), Poly-L-lysine hydrobromide (M_w_: 15000–30000), were purchased from Sigma-Aldrich (France). Rat tail type I collagen was purchased from BD Biosciences (Bedford, MA). Minimum Essential Medium Earles salts (MEM), penicillin/streptomycin, gentamicin, fetal bovine serum, HEPES buffer solution, sodium pyruvate solution, MEM Non Essential Amino Acids solution, glutamine solution, Phosphate-Buffered Saline (PBS) and trypsin-EDTA were obtained from Gibco (France).

### Animals

Female C57BL/6Jr mice (7 weeks old) were purchased from Janvier (St Berthevin, France). The mice were housed for 1 week before implantation in a climate controlled room with 12 h light/dark cycles. Experimental protocols were approved by the Animals Experiments Committee at the Direction Départementale de la Protection des Populations of Oise (C-60-60159-001). All efforts were made to minimize mice suffering.

### Solution Preparation

The Na-alg solution was obtained by solubilisation of Na-alginate (1.5% (w/v)) in a NaCl/HEPES solution (NaCl 154 mM and HEPES 10 mM, pH 7.4) and sterilized through a 0.45 µm filter. The 1% (w/v) alginate solution is obtained by adding sterile NaCl/HEPES solution to the 1.5% (w/v) alginate solution after filtration. The Na-alg/collagen solution was prepared by mixing cold type I collagen with cold sterile NaCl/HEPES solution, in an ice-bath. The sterile Na-alg solution (1.5% (w/v)) was added to the collagen one and well mixed to obtain the final concentrations of Na-alg 1% and collagen 0.045%. The Poly-L-lysine solution was prepared by solubilisation of poly-L-lysine hydrobromide (0.05%) in the NaCl/HEPES solution, sterilized by filtration (0.22 µm). The gelation solution used for all bead compositions was composed of NaCl 154 mM, HEPES 10 mM and CaCl2 115 mM, pH 7.4, and sterilized by filtration (0.22 µm). All solutions were maintained at 4°C until use.

### Bead Fabrication

The beads were prepared with the different materials listed in Table 1 using the extrusion method adapted in our laboratory [30]. Briefly, the solutions were extruded through a 24 G nozzle with a coaxial air flow. The size of the droplets was tuned by the air flow rate. The droplets fell into their respective gelation bath and were allowed to gelify for 15 min at room temperature. Then the beads were washed twice with NaCl/HEPES solution. For both bead types with a poly-L-lysine coating (compositions 2 and 4 in Table 1), the beads were incubated into a poly-L-lysine solution for 10 min. The beads were washed twice with NaCl/HEPES solution. To cover the PLL layer with a Na-alg layer, the beads were put in a Na-alg solution (0.15% (w/v)) for 4 min and after two washings in NaCl/HEPES solution, were maintained at 4°C. The size of 10 beads of each composition was measured after 1, 3, 8 and 10 days of culture with the software associated to the light microscope (Leica DMI 6000, France).

**Table 1:** Different types of material tested.

Condition	Core composition	Outside layers
1	Alginate (1% (w/v))	None
2	Alginate (1% (w/v))	PLL (0.05% (w/v)) and Alginate (0.015% (w/v))
3	Alginate (1% (w/v)) mixed with Type I Collagen (0.045% (w/v))	None
4	Alginate (1% (w/v)) mixed with Type I Collagen (0.045% (w/v))	PLL (0.05% (w/v)) and Alginate (0.015% (w/v))

### Compression Assay

The beads were subjected to a classical compression assay following the method previously described by David et al. [31]. The beads are compressed at constant speed using a computer controlled device fitted with a 2 N force transducer (Synergie 400, MTS Corporation, France). Briefly, a single layer of four to ten beads was placed on a platform submerged in NaCl/HEPES solution and compressed up to 40% of the bead initial diameter. The force exerted by the piston was recorded by the transducer. The initial contact point between the piston and the bead layer was determined with a precision of ±20 µm. The compression experiment was analyzed by means of Hertz theory [32], [33], assuming that the load was normal to the surface of the bead, that the material was elastic and obeyed Hooke’s law and that the deformation remained small. In order to get the mean force acting on each individual bead, the force measure was divided by the number of beads used for each experiment. For each bead material composition, compression tests were performed on 10 bead samples. The beads were preserved for 18 h at 4°C after manufacturing.

### Mass Transfer Kinetics of Vitamin B12

The vitamin B12 mass transfer experiments were performed under dynamic conditions following a published protocol [34], adapted from [35]. 10 mL of empty beads were located in a fluidized bed bioreactor which imposed a cyclic motion to the beads, under a perfusion flow rate of 10 mL/min. The tank concentration was continuously monitored by a parallel pump feeding the spectrophotometer cell (SPECORD, Analytik Jena, Germany). The total volume of supernatant was 24 mL. The absorbance of vitamin B12 at 360 nm was measured every 0.5 for 30 min. Equilibrium was reached before the end of the measurement time. Mass transfer coefficients were then calculated from the solute mass balance and adapted Fick’s law [34].

### Cell Encapsulation

HepG2/C3A human hepatocellular carcinoma cells, provided by the ATCC (CRL 10–741, LGC Standards Sarl, France) were cultured in MEM supplemented with penicillin (100 units/mL), streptomycin (100 µg/mL), 10% fetal bovine serum (FBS), 10 mM HEPES buffer solution, 1 mM sodium pyruvate, 1% v/v MEM non-essential amino acids and 2 mM L-glutamine. The cells were trypsinated ones a week for amplification. Before encapsulation, HepG2/C3A cells were trypsinated and detached from the culture flask, resuspended in sterile NaCl/HEPES solution and then gently mixed with the sterile Na-alg or Na-alg/collagen solution. The final cell density was 2.10^6^/mL of Na-alg. The solutions were extruded as previously described. After the encapsulation process, the beads were washed in MEM and resuspended in complete culture medium. Volumes of 400 µL of the bead solution were distributed into 24-well plates and cultured in 1 mL of culture medium for 10 days, at 37°C, 5% CO_2_. Supernatants were changed at day 2, 4, 7 and 9.

### Cell Viability

Cell viability within the beads was qualitatively assessed using fluorescence staining with propidium iodide and acridine orange (Sigma Aldrich, France) under confocal microscopy (DMI 6000 B, Leica, France). Moreover, the cell viability was quantified at day 3, 8 and 10 by the LDH released in the supernatant over 24 h. The supernatant was changed 24 h before the experiment and replaced with 1 mL of complete culture medium with 1% FBS. A mortality of 100% was considered after addition of 0.9% Triton X100 in the supernatant and incubation during 1 h at 37°C. The protocol was conducting according to the manufacturer’s recommendations (Promega, France).

### DNA Measurement

The beads were degelified in a sterile sodium citrate solution (citrate 55 mM, NaCl 154 mM) for 10 min at 37°C. After homogenization, the solution was centrifuged at 8000 g for 30 sec. The cells in pellet were washed with PBS, resuspended in 500 µL sterile milliQ water, and kept frozen at −80°C during at least one week. After thawing at room temperature, volumes of 100 µL of the cell solutions were added in a 96-well plate. Hoechst reagent (FluoReporter® Blue Fluorometric dsDNA Quantitation Kit, Molecular Probes, Invitrogen, CA, USA) was added to each well and fluorescence was measured (excitation: 350 nm, emission: 460 nm) on fluorescence spectrophotometer (SPECTRAFLUO Plus, TECAN, Switzerland). The DNA quantification was realized 1, 3, 8 and 10 days after encapsulation for each bead composition. The cell number was calculated using a standard curve correlating the final fluorescence to the C3A cell number. The calculated cell number was normalized with respect to measurement at day 1 post-encapsulation.

### Glucose Consumption

The glucose concentration in the supernatant was determined with a biochemical automatus (Konelab 20, Thermo, France) using a kit from Thermo (France). This method is based on glucose oxidase and a modified trinder color reaction, catalyzed by the enzyme peroxidase. The protocols followed the manufacturer’s recommendations. The glucose consumption was calculated over 24 h taking into account the initial glucose concentration in the medium and the cell number derived from DNA measurement assay.

### Albumin Release

Albumin concentration in the supernatant was determined by a sandwich Enzyme-Linked ImmunoSorbent Assay (ELISA) method using the Human Albumin Elisa Quantification Set (Bethyl Laboratories, USA). The protocol followed the manufacturer’s recommendations. Albumin secretion rate was calculated by dividing the amount of albumin synthesized over 24 h by the cell number derived from the DNA measurement assay.

### Bead Implantation and Histology

Female C57BL/6Jr mice were used as transplant recipients. The animals were divided in three experimental groups as followed: group I. no bead implantation; group II. implantation of Ca-alg beads (composition 1 in Table 1); group III. implantation of beads of Ca-alg mixed with collagen (composition 3). Beads were suspended in NaCl/HEPES sterile solution. 100 µl of solution containing beads were subcutaneously injected with a 25G-seringe on 8 week-old mice. The animals were sacrificed 14 days after injection. Transplanted and control skins were fixed in 4% paraformaldehyde and were processed for histology. Paraffin-embedded sections (3 µm) were stained with Hemalun Eosine Safran (HES) at the histology platform (RHEM) from Montpellier and analyzed by an anatomy pathologist.

### Statistical Analysis

Statistical analysis was performed using the One-Way Anova test followed with a Dunnett’s test to compare the differences between the modified alginate and the native one. Differences were considered to be significant when p<0.05 (*), p<0.01 (**) and p<0.001(***).

## Results

### Mechanical Properties of Cell-free Beads

#### Beads’ production

**Figure 1:**
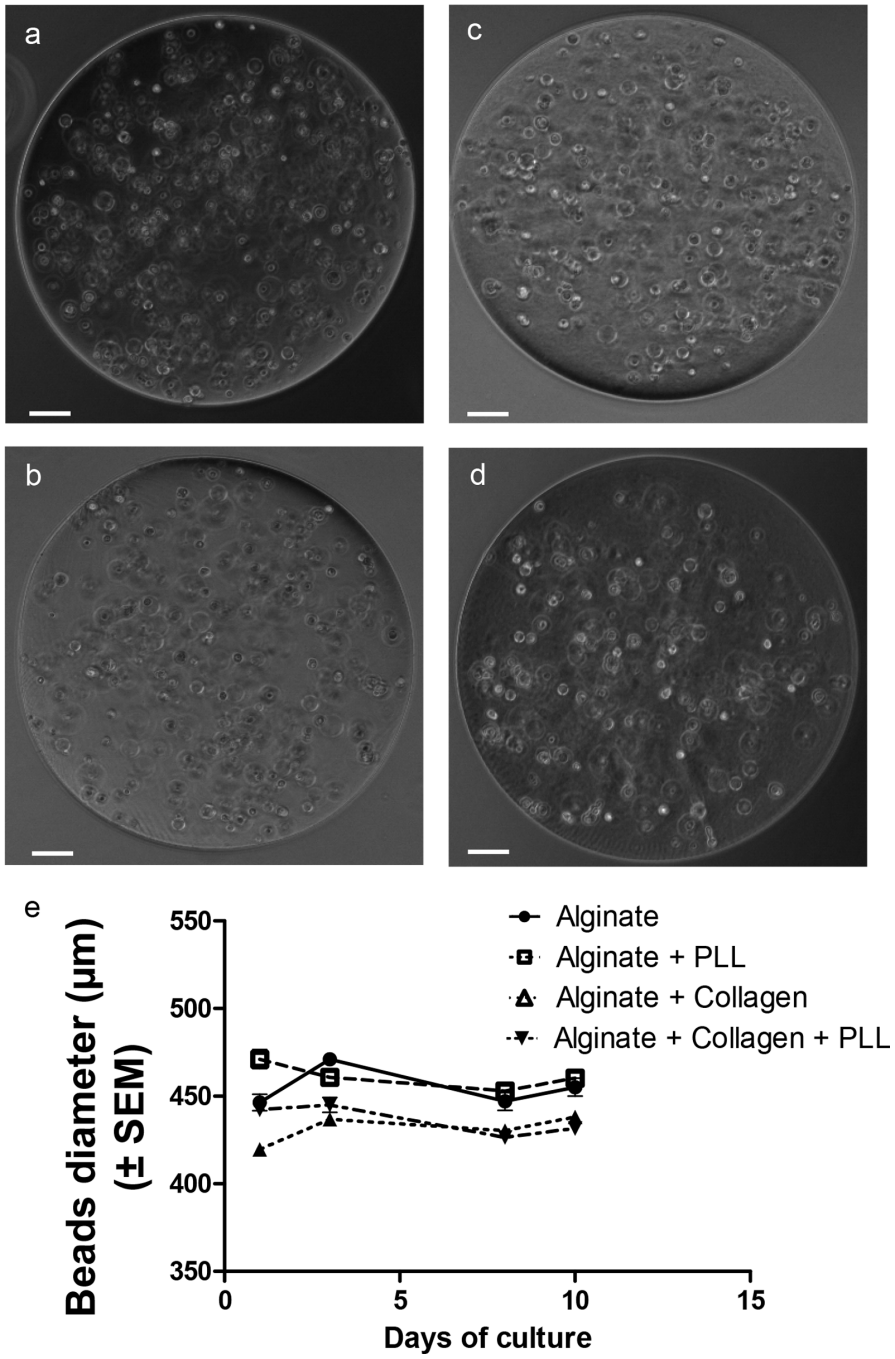
Beads obtained with the different material conditions. (a) alginate, (b) alginate with PLL and alginate layers, (c) alginate mixed with collagen, (d) alginate mixed with collagen and PLL and alginate layers. The white bars correspond to 50 µm. (e) Bead diameter evolution during the 10 days of culture.

We targeted to produce spherical and homogenous beads with a mean diameter of 450 µm, and a SD which did not overrun 5% of the mean value, showing a feasible bead production with the encapsulation device employed (Fig. 1 a to d). We observed that the mean diameters were slightly larger for Ca-alg and Ca-alg/PLL beads (455.6±21 µm and 460.7±19.6 µm, respectively) than for Ca-alg/collagen and Ca-alg/collagen/PLL beads (431,8±14 µm and 436.4±16 µm, respectively). This might be due to different interactions of alginate/collagen mixture with the gelification bath. Moreover, the bead size appeared stable during the 10 day culture (Fig. 1 e).

#### Bead mechanical properties

**Figure 2:**
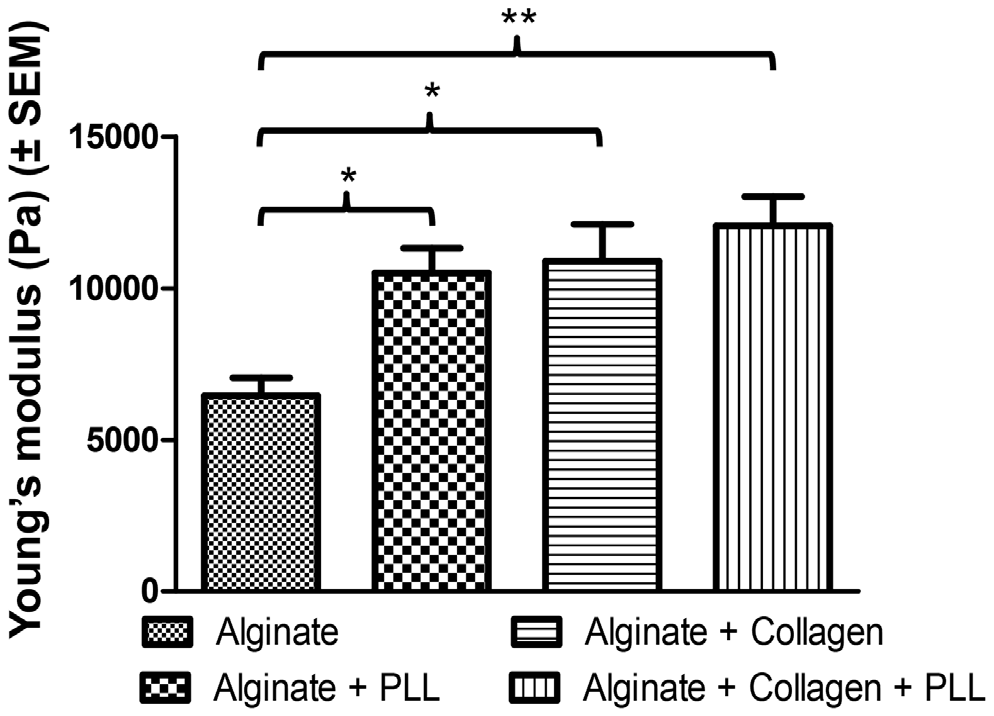
Calculated Young’s modulus for the different bead compositions. Data points represent the mean ± SEM. *p<0.05, **p<0.01. n = 10.

The Young’s modulus of the different Ca-alg beads were calculated from the measurement of the force needed to compress the beads at a constant speed up to a set percentage of their initial diameter (final height equivalent to 40% of the initial diameter). The higher the Young’s modulus, the more the material resists to compression. Our data (Fig. 2) demonstrated that the Young’s modulus significantly increased when collagen was added to Ca-alg or when the bead is coated by a PLL layer, compared to pure Ca-alg beads (1.6 fold (*p*<0.05), 1.7 fold (*p*<0.05), respectively). Moreover, the simultaneous incorporation of collagen and PLL layer resulted in a cumulative effect on the Young’s modulus (increase of 1.9 fold (*p*<0.01), in comparison with Ca-alg beads). The beads became thus much more resistant to mechanical load after enrichment with the different materials tested.

#### Vitamin B12 mass transfer

**Figure 3:**
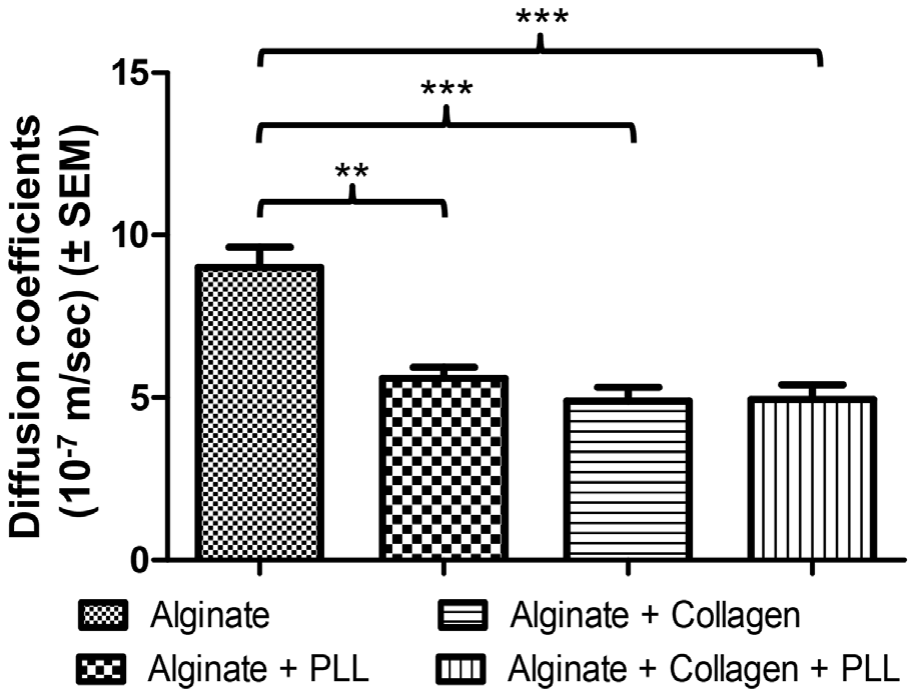
Diffusion characterization of the different biomaterials. a) Calculated diffusion coefficients. Data points represent the mean ± SEM. **p<0.01, ***p<0.001. n = 3.

The mass transfer coefficients of vitamin B12 for the different Ca-alg beads were compared to the control composition (composition 1). Adding the PLL layer or blending collagen with Ca-alg decreased significantly the bead mass transfer coefficient for the solute, compared to control (0.67 fold (*p*<0.01), 0.53 fold (*p*<0.001), respectively) (Fig. 3). Ca-alg beads with a collagen core and a PLL layer (composition 4) confirmed the significant decrease of the mass transfer coefficient compared to control composition (0.64 fold (*p*<0.001)). As the perfusion conditions and the bead sizes were identical, the differences could only be interpreted as a change in the porosity of the materials.

### Cell Viability and Proliferation

#### Cell morphology and viability

**Figure 4:**
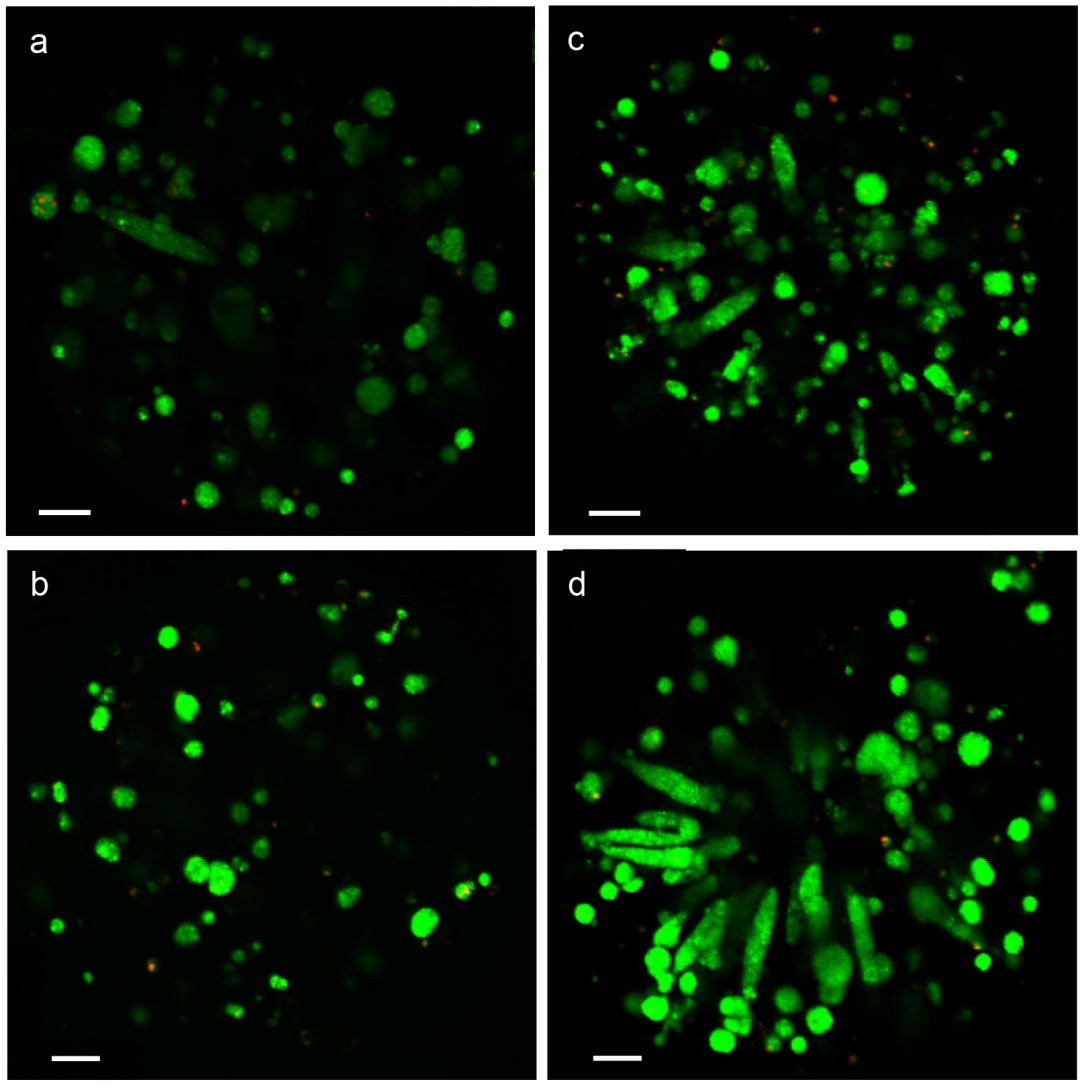
HepG2/C3A cell viability at day 8 in the different biomaterials using confocal laser scanning. (a) Alginate, (b) Alginate+PLL and alginate layers, (c) Alginate mixed with collagen, (d) Alginate mixed with collagen+PLL and alginate layers. Cells were stained with Acridine Orange (living cells, in green) and Propidium Iodide (dead cells, in red). The white bars correspond to 50 µm.

HepG2/C3A cells were encapsulated as single cells and small aggregates of 2–3 cells, with a homogeneous cell repartition in beads at day 1 (not shown). After 8 days of culture, cell proliferation was associated to 3D cell organization which adopted different morphologies, like spheroids organization in Ca-alg core compositions (compositions 1 and 2) and channel-like and spheroids organization in Ca-alg/collagen core compositions (compositions 3 and 4) (Fig. 4).

**Figure 5:**
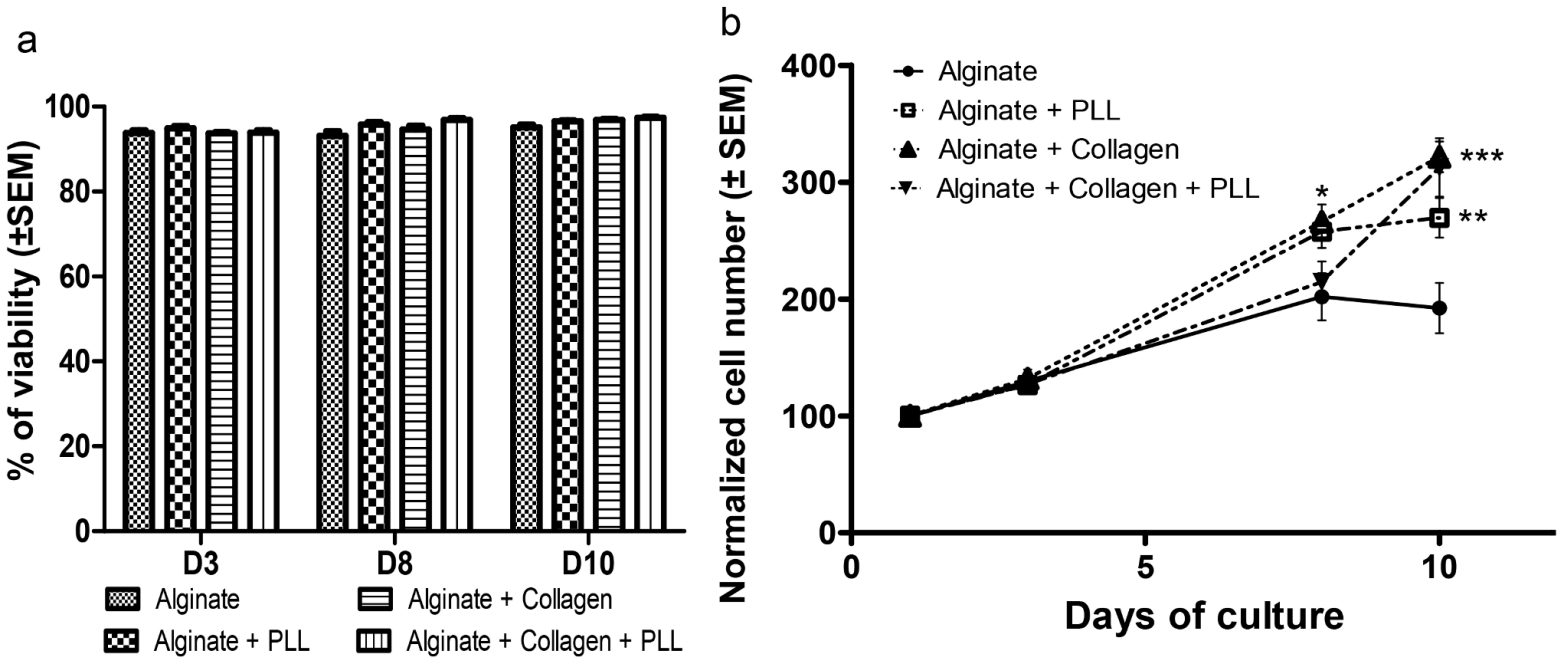
HepG2/C3A cell viability and proliferation. (a) Cell viability measured by quantification of the released LDH. The cell viability was higher than 80% from the third day of culture. (b) Cell number measured by DNA content. The DNA measurement was normalized with respect to measurement at 1 day post-encapsulation. Data points represent the mean ± SEM. *p<0.05, **p<0.01, ***p<0.001. n = 4.

The viability of the encapsulated HepG2/C3A cells was followed by acridine orange and propidium iodide stainings (Fig. 4). After 8 days of culture in static conditions, most of the cells were alive (cells in green) and only few cells were dead (cells in red), whatever the composition of the Ca-alg bead. The viability was also quantified by the LDH released in the supernatant and expressed as a percentage of viable cells. The cell viability was above 90% from day 3, whatever the material combination (Fig. 5 a).

#### Cell proliferation

The HepG2/C3A cell proliferation was measured by DNA quantification after 1, 3, 8 and 10 days of culture (Fig. 5 b). In all culture conditions, HepG2/C3A cells proliferated well, increasing their number over 10 days. If proliferation rate was not significantly different over the first 3 days of culture among the four types of Ca-alg bead compositions, the relative cell growth was significantly higher compared to Ca-alg bead, in compositions 2, 3 and 4 at day 10 (1.38 fold (*p*<0.01), 1.58 fold (*p*<0.001) and 1.60 fold (*p*<0.001), respectively).

### Functional Assays for Embedded HepG2/C3A Cells

#### Glucose consumption

**Figure 6:**
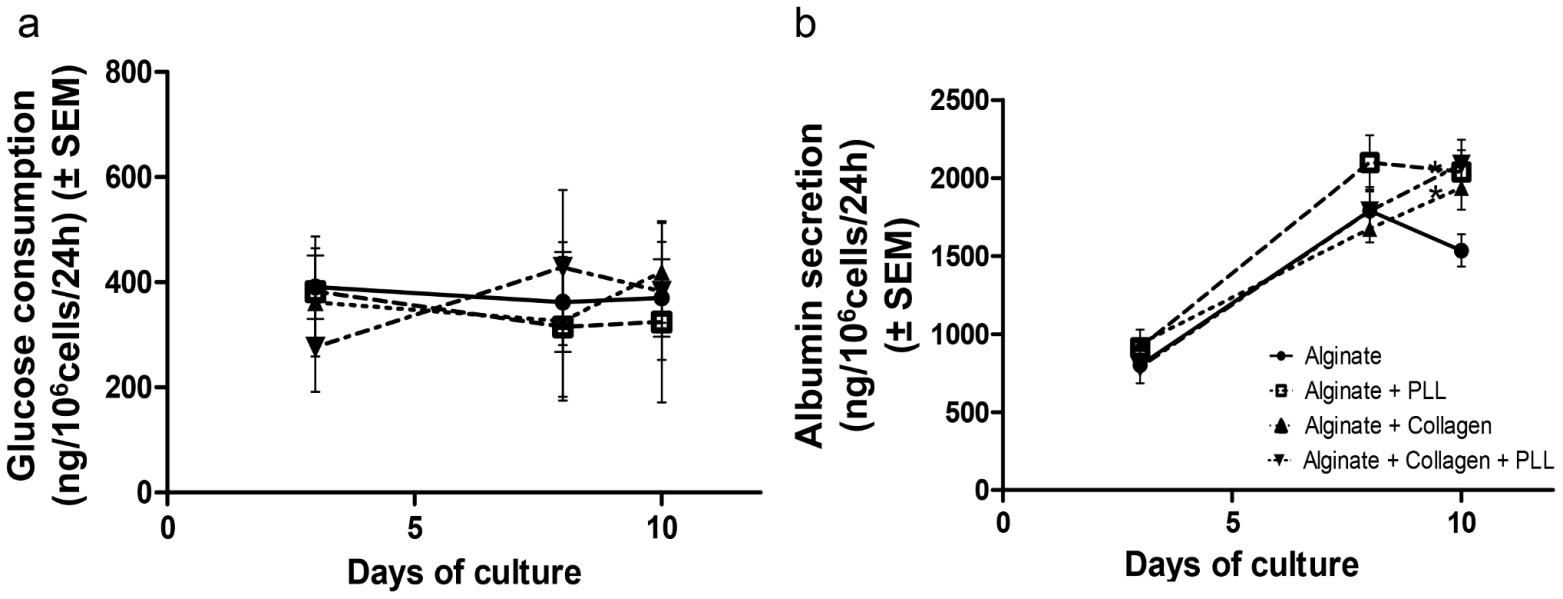
HepG2/C3A cell metabolism. (a) Glucose consumption for 1 million HepG2/C3A cells during 24 h after 3 days, 8 days and 10 days of culture in different bead compositions. (b) Albumin production during 24 h for 1 million HepG2 C3A cells entrapped in different bead compositions after 3, 8 and 10 days of culture. Data points represent the mean ± SEM. *p<0.05. n = 4.

During the 10 days of culture, the glucose consumption was stable for all compositions (around 400 ng/10^6^ cells/24 h) (Fig. 6 a).

#### Albumin secretion

As showed in Fig. 6 b, the albumin secretion increased during the first 8 days of culture in all bead compositions, varying from around 900 ng/24 h/10^6^ cells at day 1 to 1936±139 ng/24 h/10^6^ cells for the composition 3, 2038±139 ng/24 h/10^6^ cells for the composition 2 and 2086±157 ng/24 h/10^6^ cells for the composition 4 at day 10. There were no significant differences in albumin secretion at day 8 between the various Ca-alg bead compositions. From day 8 to day 10, the albumin secretion was stable in Ca-alg beads with PLL, and increased in Ca-alg beads mixed with collagen alone or combined with a PLL layer. At day 10 and compared to Ca-alg bead, the albumin secretion was higher in all the beads regardless of their composition, with a significant effect observed in compositions 2 and 4 (*p*<0.05).

### Bead Implantation

**Figure 7:**
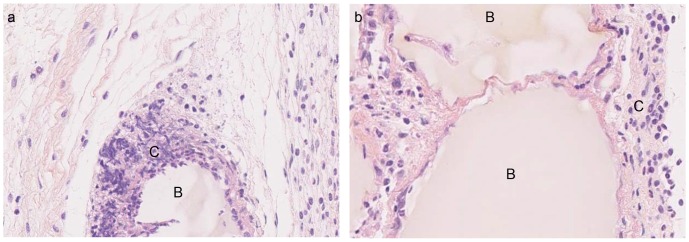
Bead implantation in mice (zoom 20x). (a) Ca-alg, (b) Ca-alg mixed with collagen. The beads were implanted during 2 weeks and the tissues were fixed and stained with Hemalun Eosine Safran (HES). B) Beads, C) Inflammatory cells.

The beads of compositions 1 and 3 were subcutaneously implanted for 14 days in immunocompetent mice, to assess the host’s biocompatibility for the control and the most promising material alteration (from the above results). The histological analysis (Fig. 7) showed an inflammation reaction (presence of inflammatory cells) around the beads in both tested compositions, which was nevertheless limited in the presence of collagen.

## Discussion

Encapsulated hepatocytes’ transplantation is potentially a successful alternative to liver transplantation. Alginate-based hepatocyte encapsulation is a well-studied subject, based on different materials to enhance bead resistance [18], material biocompatibility [11], [14], hepatocyte viability and functions [36], [37], and cell immunoprotection [38]. According to the different studies presented in the literature, several parameters appeared essential to ensure a successful implantation of cells embedded in a biomaterial:

1. Size and shape of the beads: On the one hand, the molecular exchanges between the supernatant and the core of the beads depend on the diffusion length, ie. the size of the beads [39]. On the other hand, a minimal diameter appears essential to maintain the implant at the location of the injection site. It was shown that intraportal implantation of pancreatic islets, with diameters comprised between 50 and 400 µm, permitted their engraftment in the liver [40]. As a compromise, the beads should have a similar diameter. We tested four different material combinations to obtain beads with such diameters. All of these compositions allowed spherical, homogeneous bead production, with the target diameter, as previously described in the literature [6], [11]. The bead diameters were stable and no swelling was observed over the culture period.
2. Physical properties and porosity: The biomaterial used for cell encapsulation should possess sufficient mechanical rigidity to support the blood flow rate all the time of the implantation without bead rupture and cell escape. We studied the mechanical material modifications of the beads made with different material combinations and our results showed that the external layer of PLL indeed increased the mechanical resistance of the beads by a factor of 1.6, as does the collagen inclusion in alginate. But the presence of these complexes decreased the membrane porosity and the coating limited the size of molecules diffusing into the bead [41]–[43]. As far as porosity was concerned, our data showed that the vitamin B12 diffusion coefficient decreased with the addition of PLL and alginate layers or with collagen. These results were coherent with the added rigidity probably coming from exacerbated cross linking between alginate and collagen or from the presence of two independent networks [44]. It should be noticed that the glucose consumption was stable during the culture period meaning that the diffusion of essential nutriments such as oxygen and glucose was sufficient.
3. Biological functions: It is expected that encapsulated cells are viable and functional before implantation. Our data showed the viability of the encapsulated cells was up to 80% from the third day and was maintained during the 10 days of culture. Moreover the cells proliferated in the beads regardless of their composition. These data are compatible with other one reporting that PLL layer did not limit the encapsulated hepatic cell proliferation [45]. Cell-cell contacts are essential for hepatocyte viability and functionality. The 3D microenvironment offered by the bead matrix allowed cell-cell interactions resulting in multicellular aggregates with a structural organization mimicking the hepatic tissue. The 3D cell organization was different in the tested compositions. Our data showed a spheroid organization in alginate core beads. When collagen was mixed to alginate, we observed an increase of the cell channel-like organization (Fig. 4). The same behaviors for cell reorganization have been observed in the literature for hepatic cell-lines encapsulated in alginate [46], [47]. Moreover, due to a better interaction between the cells and the biomaterial, HepG2/C3A cell proliferation was more important in the compositions where collagen was mixed to alginate. The different bead compositions exerted here a relative influence on the kinetics of albumin release, with a maximal value obtained 8 days after the encapsulation step as it was noted by Khalil et al. [26]. When encapsulated in collagen beads, HEPG2/C3A cells improved albumin secretion, confirming the importance of cell-matrix components interactions in the maintenance of liver-specific functions [48]. Depending on the type of collagen, this influence could be optimised [49]. The rate of albumin synthesis observed in our culture model has to be considered for transplantation tests as a gauge of hepatic anabolism status. In animal models, serum albumin decreased after acute liver failure induction, but stably returned to normal level when microencapsulated hepatocytes are transplanted [50]. This therapeutic effect, associated to a better survival rate, can be enhanced by transplantation of co-microencapsulated hepatocytes and islets of Langerhans, human umbilical vein endothelial cells or stromal cells [51], [52].

Table 2 summarizes the different requirements and the effects of the different material compositions on both physical and biological properties. It appears that the best compromise between mechanical strength and cell behavior is an alginate-collagen core with or without layers of PLL and alginate.

**Table 2:** Effects of the different compositions on the analyzed parameters.

	Alginate	Alginate+PLL	Alginate+Collagen	Alginate+Collagen+PLL
Bead fabrication (400 µm)	+++	+++	+++	+++
Mechanical strengh	+	++	++	+++
VitB12 mass transfer	+++	++	++	++
Cell viability in culture at D10	+++	+++	+++	+++
Cell proliferation at D10	+	++	+++	+++
Glucose consumption at D10	+++	+++	+++	+++
Albumin secretion at D10	+	+++	++	+++

In order to assess the host’s response in case of *in vivo* implantation of alginate beads, we subcutaneously implanted cell-free beads in mice for two weeks. We chose to implant only beads with compositions 1 and 3. Pure alginate beads (composition 1) were used as a control [11]. In both cases, tissues did not appear fibrotic after 14 days, but an inflammatory response was observed by histology, although reduced in the case of alginate mixed with collagen. The same results were shown in the literature for the same type of alginate with a high content of M-blocks [11] and for Ca-alg mixed with collagen beads [6]. This composition seems suitable to implant bead-encapsulated cells. However, the results must be confirmed when the beads are implanted in the sites the most adapted to treat hepatic failures.

### Conclusion

This comparative study was conducted to evaluate the influence of various Ca-alg based matrix composition on cell encapsulation process, mechanical resistance, diffusion properties of vitamin B12, biocompatibility and cell behavior of encapsulated HepG2/C3A cell line, in order to select the best biomaterial candidates for implantation. The current data showed that PLL layer and collagen mixed to Ca-alg permitted to increase the beads’ resistance. The addition of collagen enhanced encapsulated hepatic cell line behavior. Moreover, the implantation data demonstrated that collagen mixed to alginate reduced inflammatory response. In conclusion, the best compromise between mechanical strength, cell behavior and biocompatibility is an alginate-collagen core with or without layers of PLL and alginate. The next studies performed *in vivo* should bring more conclusions about the best choice for bead implantation in the case of liver failure.
